# Cognitive Performances Are Selectively Enhanced during Chronic Caloric Restriction or Resveratrol Supplementation in a Primate

**DOI:** 10.1371/journal.pone.0016581

**Published:** 2011-01-31

**Authors:** Alexandre Dal-Pan, Fabien Pifferi, Julia Marchal, Jean-Luc Picq, Fabienne Aujard, on behalf of RESTRIKAL Consortium

**Affiliations:** 1 Mécanismes Adaptatifs et Evolution, UMR 7179 Centre National de la Recherche Scientifique, Muséum National d'Histoire Naturelle, Brunoy, France; 2 Laboratoire de Psychopathologie et Neuropsychologie, UFR Psychologie, Université Paris 08, St Denis, France; Université Pierre et Marie Curie, France

## Abstract

Effects of an 18-month treatment with a moderate, chronic caloric restriction (CR) or an oral supplementation with resveratrol (RSV), a potential CR mimetic, on cognitive and motor performances were studied in non-human primates, grey mouse lemurs (*Microcebus murinus*).

Thirty-three adult male mouse lemurs were assigned to three different groups: a control (CTL) group fed *ad libitum*, a CR group fed 70% of the CTL caloric intake, and an RSV group (RSV supplementation of 200 mg.kg^−1^.day^−1^) fed *ad libitum*. Three different cognitive tests, two motor tests, one emotional test and an analysis of cortisol level were performed in each group.

Compared to CTL animals, CR or RSV animals did not show any change in motor performances evaluated by rotarod and jump tests, but an increase in spontaneous locomotor activity was observed in both groups. Working memory was improved by both treatments in the spontaneous alternation task. Despite a trend for CR group, only RSV supplementation increased spatial memory performances in the circular platform task. Finally, none of these treatments induced additional stress to the animals as reflected by similar results in the open field test and cortisol analyses compared to CTL animals.

The present data provided the earliest evidence for a beneficial effect of CR or RSV supplementation on specific cognitive functions in a primate. Taken together, these results suggest that RSV could be a good candidate to mimic long-term CR effects and support the growing evidences that nutritional interventions can have beneficial effects on brain functions even in adults.

## Introduction

With the increase of human longevity have appeared a significant number of age-related diseases and more particularly, age-related brain dysfunctions. Thus, a growing interest for non-genetic anti-aging strategies development as food protocols is revealed. Caloric restriction (CR) is the only non-genetic manipulation known to increase longevity and delay age-related diseases in various species, including mammals (see [1] for review). Interestingly, CR protocol was able to improve memory performances in healthy elderly humans [2]. However, given that CR is difficult to implement in humans due to social and practical constraints, development of CR mimetic compounds might be a suitable alternative. In the last five years, numerous studies focused on the development of these CR mimetic compounds [3], [4], [5], [6]. Among them, resveratrol (RSV), a natural polyphenolic compound that activates proteins implicated in energy metabolism homeostasis, seems to be a promising anti-aging molecule [7], [8], [9], [10], [11] which could slow brain functions decline with age.

Many studies have focused on long-term effects of CR in rodents but few data are available about RSV effects. CR treatments were mainly initiated early in the life of the animal (young or adult age) to observe the effects at a later age. It was demonstrated that CR started at mid-life in adult mice allowed to preserve strength, coordination and spontaneous alternation behaviour when the mice began to age [12]. Conversely, some studies in rats reported that CR protocol began at young age failed to provide protection against deficits in cognitive tasks in aged animals [13]. Yanai et al. demonstrated that CR has even negative effects on cognitive performance in a spatial discrimination task in aged rats submitted to moderate CR throughout their life [14]. A possible cause of such a cognitive decline was the lower availability of energy substrates to the brain [14]. In monkeys (*Macaca mulatta*), preliminary results demonstrated that aged primates exhibited a reduction of age-related diseases and brain atrophy after several years of CR [5], [15]. However, its impact on cognitive functions remains unknown in this non-human primate. Moreover, these results were observed in aged monkeys but response in adults remains unknown. Several other studies have been developed directly in older individuals to assess the short-term effects of CR on cognitive performances. Witte et al. demonstrated in elderly Human volunteers that a 3-month moderate CR induced a significant increase of 20% in verbal memory scores, which was correlated with decreases in fasting plasma levels of insulin [2]. Moreover, a 10-day CR in 24-month old rats can suppress age-related increase in oxidative stress and inflammatory process, two major causes of brain dysfunctions [16]. To our knowledge, only one study demonstrated that adult rodents subjected to a 6-month diet restriction protocol presented better learning and consolidation processes [17] and no study implemented in a nonhuman primate has demonstrated the effects of long-term CR in adult animals. Several studies have demonstrated the role of RSV in maintaining neuronal function in rodents (see [18] for review). RSV was able to prevent cognitive impairment in rat submitted to artificial treatments inducing Parkinson's disease [19], diabetes [20], neuroinflammation [21], head injuries [22] or in the case of transgenic rodent models of Alzheimer's disease [23]. To our knowledge, only one study on a fish showed a protective effect of RSV against cognitive decline due to natural aging [24]. However, rigorous assessments of RSV effects on normal cognitive function in adult nonhuman primates remained to be performed.

One originality of the present study lies on the animal model, the grey mouse lemur (*Microcebus murinus*) which is a nocturnal prosimian primate originating from Madagascar with a life expectancy of 8–10 years. The grey mouse lemur presents specific characteristics for a primate that makes it a unique model to assess the effects of CR or RSV treatments on a non-human primate organism. Besides practical aspects related to its small size (around 15 cm without tail) and its low body mass (80 to 120 g), the mouse lemur has the ability to make energy reserve once a year during the winter season thanks to important fattening process [25], [26]. Furthermore, this animal presents a daily hypothermia phase allowing to adjust the energy reserves in accordance with environment constraints [27], [28]. Previous study has already shown that CR or RSV supplementation modified the regulation of energy balance in mouse lemur [29] but no data was provided about the effects of these treatments on cognitive performances of these animals. Yet, comparable to what is observed in humans, cognitive impairments [30] and MRI-evaluated cerebral atrophy [31], [32] were demonstrated in this primate. We can hypothesize that CR or RSV supplementation would improve the cognitive functions of adult mouse lemurs after several months of treatment.

In the present study, we assessed the effects of a moderate chronic CR or RSV supplementation, initiated at adult age (38±2 months), on cognitive and motor functions in mouse lemur. We used three different tasks: (i) the continuous spontaneous alternation (CSA) task which involves working memory managed by striato-prefrontal circuits, (ii) the circular platform (CP) task which requires hippocampal system-dependent spatial memory and (iii) the conditioned place preference (CPP) task which assesses emotional memory mediated by the amygdala. Three criterions were considered for selecting these cognitive tests: (i) age-related impairment on these tests has been reported in various species [33], [34], [35], (ii) the different tests presumably tax different cognitive capacities subserved by different cerebral structures [36] and, (iii) no food reinforcement is required and, consequently, motivation to achieve these tasks is independent of diet. Besides cognitive functions, motor abilities were evaluated using the Rotarod test and a jump test completed with spontaneous locomotor activity measurements. Emotionality was assessed using the open field task. In order to evaluate the impact of both treatments on stress, urinary cortisol level was also assessed. Physical fitness and emotionality were measured as to make sure that differences in performance on cognitive tasks did not rely on non-cognitive functions.

The objective of our study was to evaluate and to compare, for the first time, the impact of a chronic and moderate CR or mimetic compound (RSV) supplementation on cognition and motor performances in adult primates. We expect better cognitive and motor performances of CR group compared to control group after 18 months of treatment. Moreover, we hypothesize that RSV supplementation will mimic observed CR effects by improving cognitive and motor functions of grey mouse lemurs. These issues are of major importance in the validation and implementation of such long term protocols in humans.

## Materials and methods

### 1 Ethics Statement

All experiments were performed in accordance with the *Principles of Laboratory Animal Care* (National Institutes of Health publication 86-23, revised 1985) and the European Communities Council Directive (86/609/EEC). The Research was conducted under the authorization n° 91–305 from the “Direction Départementale des Services Vétérinaires de l'Essonne” and the Internal Review Board of the UMR 7179. All the experiments were done under personal license (authorization number 91–460, issued 5 June, 2009) delivered by the Ministry of Education and Science. In accordance with the recommendations of the Weatherall report, “The use of non-human primates in research”, special attention was paid to the welfare of animals during this work [37]. All efforts were made to minimize nociception.

### 2 Subjects and dietary interventions

All the male grey mouse lemurs (*Microcebus murinus*, Cheirogaleidae, primates) used in this study were part of the Restrikal study described previously [29]. In the breeding colony, animals are exposed to an artificial photoperiodic regimen consisting of six months of summer-like long day length (14∶10 h light-darkness) and six months of winter-like short day length (10∶14 h light-darkness). Analysis of survival from 254 male mouse lemurs from the captive colony allowed to determine the mean life span (72±2 months), the mean life span of the 10% of the most long lived animals (120±2 months) and the observed maximal survival duration (144 months which corresponds to 12 years). Thirty-three animals were used. Animals were included at the age of 38±2 months, at the onset of their winter-like season (short days, SD). Animals were weighed at the beginning of the experiment (87±3 g) and during each test session to monitor body mass variations.

Dietary protocol of the Restrikal study has already been described [29]. Briefly, animals were fed with fresh fruit and a daily mixture made up of cereals, milk and egg. Water was always given *ad libitum*. Animals were randomly assigned to each experimental group. Three different groups were formed: an *ad libitum* control group (CTL) of 11 animals, a CR group of 10 animals which was fed with the same diet but received 30% less than CTL and, a third RSV group of 12 animals which was fed with the same quantity of food as CTL but supplemented with 200 mg of RSV per kilogram body weight per day (Sequoia Research Products, United Kingdom). In order to know the exact quantity of food ingested by the animals, daily leftover were measured and corrected for water evaporation.

Cognitive and motor tests were performed at the end of the SD period because it corresponds to one of the most important active phases in grey mouse lemur. All tests were realized during the last 6 hours before their daily active phase. Thereby, cognitive tests were performed after 18 months of treatment (animals were 56±2 months) and the animals presented a mean body mass of 126±26 g for CTL, 112±30 g for CR and 129±30 g for RSV. No statistical difference was observed for the mean body mass between groups (CTL vs CR, U = 47.5, p = 0.597 and, CTL vs RSV, U = 59, p = 0.667).

### 3 Continuous spontaneous alternation (CSA) task

#### 3.1 Apparatus

The test was performed in a plus-maze constructed of wood (each arm: 25 cm high×40 cm long×15 cm wide). The maze rested on papers that were changed between trials. The four arms (labelled A, B, C and D) ended with 90° left turns (10 cm long). Thus, the ends of the arms were not visible from the centre of maze and, as a consequence, had incentive effect on mouse lemur exploratory behaviour. In order to avoid jumps over the walls of the maze, a one-way mirror covered the top of the maze. This ceiling permitted observation by experimenter but prevented mouse lemurs from seeing extramaze cues. Different intramaze cues such as pieces of plastic, foam rubber or cardboard were placed on the walls of each arm in order to differentiate them. A red 15 W bulb was placed halfway on the top of the longer wall of each arm and provided the only light in the room during testing.

#### 3.2 Testing procedure

At the beginning of the trial, the animal was placed in the centre of the maze with all the four alleys shut off by opaque doors. After 30 seconds, the doors were slowly raised and the mouse lemur was allowed to explore freely the four arms for 20 minutes. The number and the sequence of entries (all four paws into a given arm) were recorded. The latency before the first entry was also noted (expressed in sec). Partial alternation was defined as entry into three different arms on the same overlapping sets of four consecutive choices. Total alternation was defined as entry into four different arms on the same overlapping sets of four consecutive choices. For example, a set consisting of arm choices B, D, C, B, was considered an alternation. The possible alternation sequences are equal to the number of arms entries minus three. The percent alternation is equal to the ratio of (actual alternation/possible alternation) X 100. Values were expressed as percentage. Only data from animals that made at least 6 arm entries were included in the behavioural analyses. N = 6 CTL, n = 6 CR and n = 9 RSV animals succeeded this test.

### 4 Circular platform (CP) task

#### 4.1 Apparatus

The CP task apparatus was an adaptation for mouse lemurs of the device described by Barnes [38]. It consisted of a white circular platform (diameter, 100 cm) with 12 equally spaced circular holes (each 5 cm in diameter) located 3 cm from the perimeter. The platform could be rotated. The maze platform was affixed 60 cm above the floor, and a cardboard nestbox (10 cm×10 cm×20 cm) could be inserted and removed beneath each hole and served as a refuge (goal box). A black, small plywood box could be slid beneath the non-goal holes to stop the lemurs from jumping through these holes while permitting head entering. To prevent the mouse lemur from escaping, the platform was entirely surrounded with a white wall 25 cm high across its circumference and covered with a transparent Plexiglas® ceiling that permitted the mouse lemurs to see the extra-maze visual cues. The apparatus was surrounded by a black curtain hung from a square metallic frame (length of the side, 120 cm) located 110 cm above the floor. The center of the frame was a one-way mirror to allow observation. Affixed beneath the one-way mirror and following the circular perimeter of the maze (about 50 cm above the platform) were 24 2-W lights evenly spaced, illuminating the maze. Between the one-way mirror and the upper edge of the wall, various objects were attached along the inner surface of the curtain to serve as visual cues. The starting box was an open-ended dark cylinder positioned in the center of the platform. Transparent radial Plexiglas partitions (25 cm high×20 cm long) were placed between the holes to prevent the strategy used by some mouse lemurs to go directly to the periphery of the platform and then walk along the barrier wall and inspect each hole one by one. Consequently, animals had to return to the center of the platform after each hole inspection.

#### 4.2 Testing procedure

Animals were given one day of habituation and training (day 1) and one day of testing (day 2). Each day comprised four trials, each of which began with placement of the animal inside the starting box. After 30 seconds, the box was lifted to release the animal. For the lemurs, the objective was to reach the goal box positioned beneath one of the 12 holes, kept constant in the room for all trials. When the animal entered the goal box, the trial was stopped, and the animal was allowed to remain in the goal box for 3 minutes. After each trial, the platform was cleaned and randomly rotated on its central axis to avoid the use of intra-maze cues, although the position of the goal box was kept constant in the room.

On day 1, trials 1 and 2 consisted of placing the animal in a four-walled chamber containing only the opened goal hole (one-choice test). For trials 3 and 4, the platform comprised six evenly spaced open holes (six-choices test). These two trials permitted the animal to explore the maze, observe the visual cues, and further learn the position of the goal box.

On day 2 (testing day), 12 holes were opened during the four trials. Performance was assessed by the time required for the animal to reach the right exit (expressed in sec) and the number of errors prior to reaching the goal box. An error was defined as an inspection made by inserting the nose into an incorrect hole. Thus, values were expressed in error numbers. Only data from animals that reached the goal box before 30 min of testing were included in the behavioural analyses. N = 8 CTL, n = 9 CR and n = 8 RSV animals succeeded this test.

### 5 Conditioned place preference (CPP) task

#### 5.1 Apparatus

The apparatus designed for the CPP task consisted of two chambers made of wood and that differed in shape and in wall and floor covering (foam rubber, newspapers, cardboard, plastic, etc.). One chamber was rectangular (33 cm long×26 cm wide×20 cm high) and the other was triangular (35 cm base×45 cm depth×20 cm high). Each chamber was lit by a red 15 w bulb. A small window made of one-way mirror was inserted in the ceiling of each chamber providing the experimenter the inside view of the chamber. During pre-exposure and preference phases, the two chambers were connected by a transparent Plexiglas cylinder (30 cm long×10 cm diameter). The entire apparatus was placed on a wood table 100 cm above the floor. A red 15 w bulb was placed 30 cm above the Plexiglas cylinder enabling the mouse lemur to see the outside and to be visible from the outside. This relied on the natural tendency of mouse lemurs to avoid open spaces and prevented the mouse lemur from staying too long into it.

#### 5.2 Testing procedure

The test took place over six consecutive days and consisted of three discrete phases: (i) pre-exposure phase, (ii) conditioning phase, (iii) test phase.

On pre-exposure phase (day 1), the animal was placed inside the cylinder and thereafter the cylinder was fixed between the two chambers. After 30 seconds, the sliding doors that blocked the ends of the cylinder were removed, allowing the animal to freely access to both chambers for a 15-min period. This exploration period started when the mouse lemur entered a chamber. Time spent in each of the two chambers was recorded. Animal was considered to be in a chamber when both its four paws were in it. No reinforcing stimulus was associated with either of the chambers during this phase. This phase provided the mouse lemur with some experience of both contexts and enabled the experimenter to determine which are the preferred and the non-preferred chamber for each animal. The preferred chamber was determined as the chamber in which the animal stayed at least twice the time compared to the other chamber.

On conditioning phase (days 2–5) mouse lemurs were confined to each chamber on alternate days for a 30-min session. During the days 2 and 4, each animal was placed inside its preferred chamber. Aversive stimuli associated with this chamber were a white bright light and a brief shaking of the chamber at 5^th^, 15^th^ and 25^th^ minute. This chamber was referred to as the negative paired chamber. During the days 3 and 5, each animal was placed inside its non-preferred chamber. The rewarding stimuli associated with this chamber were a dim red light and, after 15 minutes, the introduction of the own nest box of each animal. This chamber was referred to as the positive paired chamber.

On test phase (day 6), each mouse lemur was given free access to both chambers for a 15-min period and the time spent in each chamber was recorded, in a similar way to that used during the pre-exposure phase. Thus, we could estimate the percentage of reversal by subtracting the percentage of time spent in the preferred chamber by the animal on day 6 to those obtained in day 1. Values were expressed in percentage. Only data from animals for which we could determine a preferred chamber during day 1 were included in the behavioural analyses. N = 8 CTL, n = 8 CR and n = 9 RSV animals succeeded this test.

### 6 Open field task

#### 6.1 Apparatus

This system was an open-field consisting of bright and opaque Plexiglas® wall (100×100×20 cm) and covered with a transparent Plexiglas® ceiling. Four white lights of 15 W were placed at each corner of the system. The open field session was recorded by camera and the data were analyzed after the session, which avoided the presence of an observer in the room during the test.

#### 6.2 Testing procedure

The mouse lemurs were placed in an open-field for free exploration for 30 min. At the end of the session, the nestbox of the mouse lemur was placed in a corner of the open field (the same corner for all animals) to allow him to return to its nestbox with a minimal stress.

Because of persistent immobility, peripheral tracking and limited exploration are index of stress and anxiety in mouse lemurs when placed in a novel environment. We determined two parameters reflecting the degree of emotionality for each animal: (i) latency of the first movement to explore the field (expressed in sec) and (ii) total distance performed by the animals (expressed in cm). Data from animals that did not move during the 30 minutes of the experiment were excluded of the behavioural analyses. N = 9 CTL, n = 9 CR and n = 10 RSV animals succeeded this test.

### 7 Accelerating rotarod task

#### 7.1 Apparatus

This apparatus allowed quantification of fine motor coordination and balance by measuring with a chronometer, the amount of time that a mouse lemur could remain standing on a rotating, accelerating rod (model 7750, Ugo Basile, Italy). The rod was a plastic drum, 5 cm in diameter, which was machined to provide traction. The rotational speed of the system could progressively increase to up to 40 rpm.

#### 7.2 Testing procedure

The animal was placed on the rotating cylinder at 20 rpm. The rod then accelerated steadily until the end of the test which was reached when the animal fell or gripped on the rod during at least three consecutive turns without stabilizing its balance. Latency to fall or grip on the rod was recorded for each trial. Animals underwent 5 consecutive trials and the best result was recorded. Values were expressed in seconds. The system was cleaned between each trial. Data from animals that jumped from the apparatus were excluded of the behavioural analyses. N = 7 CTL, n = 9 CR and n = 8 RSV animals succeeded this test.

### 8 Jump task

#### 8.1 Apparatus

The test was performed in a closed chamber of wood (200 cm high×50 cm long×50 cm wide). The apparatus was affixed 30 cm above the floor. A one-way mirror covered one side of the closed chamber that permitted observation by experimenter without disturbing mouse lemurs. In the close chamber, an adjustable metal rod was installed that allowed to progressively increase the height of this rod of 10 cm between each effective test. A hatch located at the base allowed to introduce the animal in the closed chamber. To motivate the animal to jump, a 20 W bulb was placed below the system and its nestbox was installed 10 cm above the metal rod.

#### 8.2 Testing procedure

The animal was introduced in the closed chamber for a test of maximum 5 minutes. For the first trial, the metal rod was placed at a height of 20 cm. When the animal succeeded to rejoin its nestbox, the trial was stopped, and the animal was allowed to remain in its nestbox for 1 min. The height of the metal rod was raised by 10 cm between each successful trial. If the animal did not reach its nestbox after 5 minutes, the test was stopped and the animal was manually removed from the apparatus. The maximum height (cm) reached by the mouse lemur was noted. The system was cleaned between each trial. Data from animals that did not jump after 5 min were excluded of the behavioural analyses. N = 9 CTL, n = 11 CR and n = 10 RSV animals succeeded this test.

### 9 Spontaneous locomotor activity

Animals were housed individually in a laboratory-made locomotor activity cage with a capacity of 1 m^3^ each provided with nest and supports. Spontaneous locomotor activity was estimated using a system of presence and motion sensors placed in the cage and the nest created by R. Botalla and adapted to the mouse lemur. Presence sensors (Honeywell – transmitter: SEP8705003, receiver: SDP8405014) were placed on both sides of the nest and were continuously recording in order to detect animal presence in the nest. Two motion sensors (GARDTEC – Gardscan ‘M’ series infra-red detectors) were placed in the corners of the cage to detect the spontaneous movements of the animal. During animal movements the motion sensors recorded data every two seconds. Data were stored in a computerized system (developed in the laboratory by R. Botalla). They were then computed to represent time-course of these movement patterns using a software filtering “ACTOCEBE 3.0” developed in language G from National Instruments (software created by R. Botalla). Based on animal activity, total movements were averaged on 5 minutes intervals for further analysis and were expressed in arbitrary unit (a.u.). In the present study we focused on night locomotor activity (mean locomotor activity during the night) which is the active period of Grey mouse lemurs. Spontaneous locomotor activity was measured during 14 consecutive days for each animal. N = 7 CTL, n = 6 CR and n = 7 RSV supplemented animals were submitted to the locomotor activity measurement.

### 10 Urinary cortisol level assessment

To avoid the handling stress during urinary cortisol assessment, urine samples were collected overnight using a urine collector with ice placed below the cage of the animals. Urine samples were stored at −20°C until assayed. Urinary cortisol concentrations were measured twice on 10 µl of urine by an immunoenzymoassay (Demeditec Diagnostics, Germany). The minimum detectable cortisol concentration was 10 ng/ml. The mean intra- and inter-assay coefficients of variation were 7.8% and 7.4%, respectively. To control the variation in volumes and concentrations of the voided urine, the creatinine (Cr) content of each urinary sample was determined with a Cr colorimetric test (Quidel Corporation, San Diego, CA, USA). Urinary cortisol concentrations were expressed as microgram per gram of Cr.

### 11 Data analyses

All values are expressed as mean ± SEM. Only animals that succeeded a test were taken into account in our analyses, what explained the different numbers of animals followed in the tests. The mean failure rate was 22±3% and was independent of treatment. Mann-Whitney U test was used to assert significant variations between the CTL group and CR or RSV supplemented animals in all studied parameters. Spearman correlation analyses were performed to determine the degree and direction of association between body mass and physical performances of the animals. Comparisons were considered to differ significantly when p<0.05. All statistical computations were performed with SYSTAT for Windows (V9, SPSS Inc., USA).

## Results

### 1 CSA task

**Figure 1:**
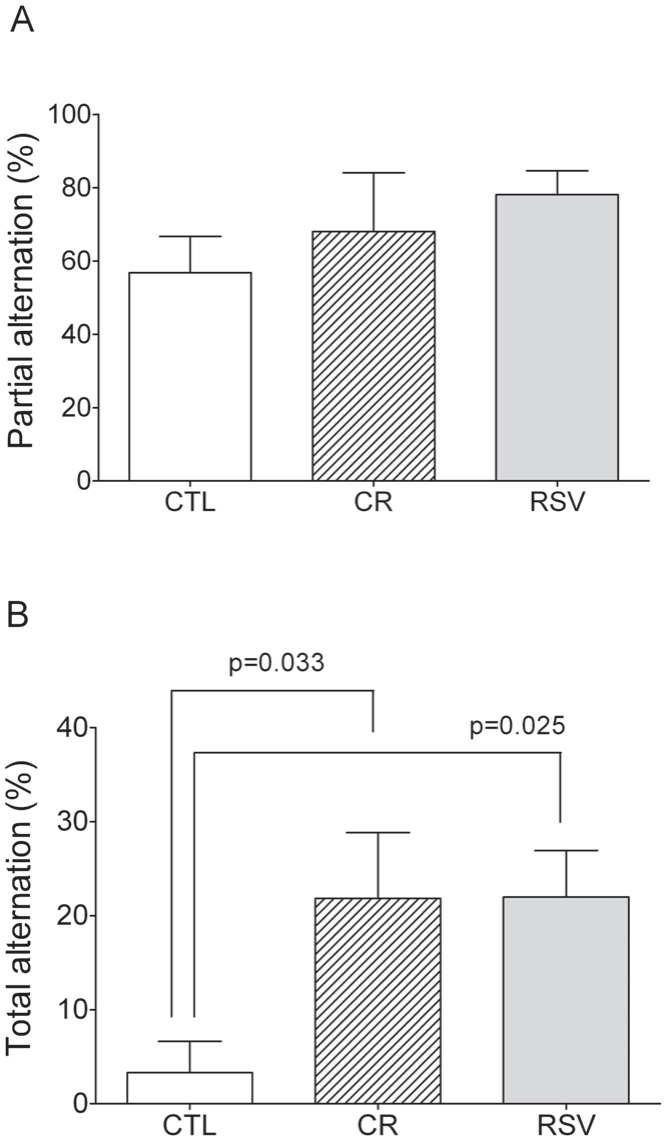
Effects of CR and RSV treatments on cognitive performances in a continuous spontaneous alternation (CSA) task. Figure 1A and B respectively represents the partial and total alternation (%) in the CSA task of control (CTL, n = 6), caloric restricted (CR, n = 6) or resveratrol supplemented (RSV, n = 9) mouse lemurs after 18 months of dietary interventions. Values are mean ± SEM.

No significant effect of both treatments was found for the latency of first movement in the CSA task which averaged 73±20 s (CTL vs CR, U = 28, p = 0.581; CTL vs RSV, U = 33.5, p = 0.856). Similarly, no significant difference appeared between the three groups for the number of corridors visited with an average of 18±3 corridors in each group (CTL vs CR, U = 63, p = 0.323; CTL vs RSV, U = 29.5, p = 0.074). No significant effect was observed between CR *vs* CTL (68±16% and 57±10%, respectively, U = 23.5, p = 0.377) and RSV *vs* CTL (78±7% and 57±10%, respectively, U = 14.5, p = 0.140) for partial alternation (Fig. 1A). Total alternation is very low in the CTL group (3±3%) and was significantly higher in CR animals (22±7% of total alternation) compared to CTL (U = 30.5, p = 0.033). Similarly, RSV fed animals had significantly higher total alternation (22±5%) compared to CTL animals (U = 9, p = 0.025, Fig. 1B).

### 2 CP task

**Figure 2:**
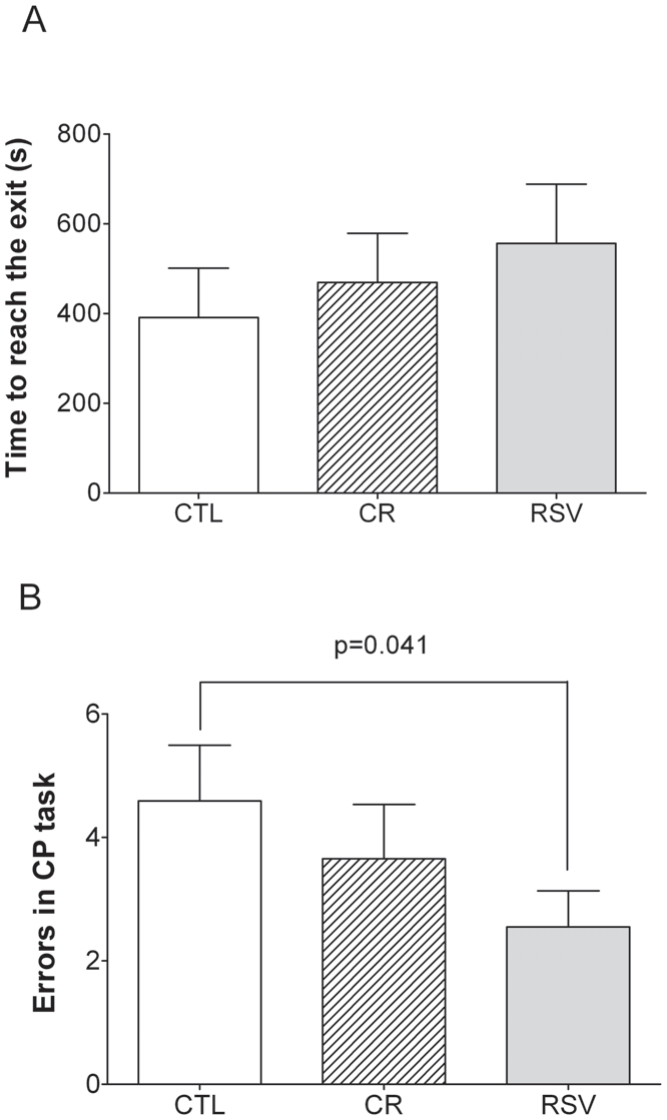
Effects of CR and RSV treatments on cognitive performances in a circular platform (CP) task. Figure 2A and 2B respectively represent the time (expressed in sec) to reach the exit and the number of errors before succeeding to find the exit of control (CTL, n = 8), caloric restricted (CR, n = 9) or resveratrol supplemented (RSV, n = 8) mouse lemurs after 18 months of dietary interventions. Values are mean ± SEM.

No significant treatment effect was observed concerning the time required for the animals to reach the exit in the CP task (Fig. 2A, CTL vs CR, U = 51.5, p = 0.910; CTL vs RSV, U = 58; p = 0.895) which averaged 480±68 s. RSV supplementation had a significant effect on the number of errors made in CP task compared to CTL animals (3±1 errors in RSV supplemented animals, 5±1 in CTL animals, U = 91, p = 0.041). No significant effect of CR was observed (4±1 errors, U = 29.5, p = 0.120) (Fig. 2B).

### 3 CPP task

**Figure 3:**
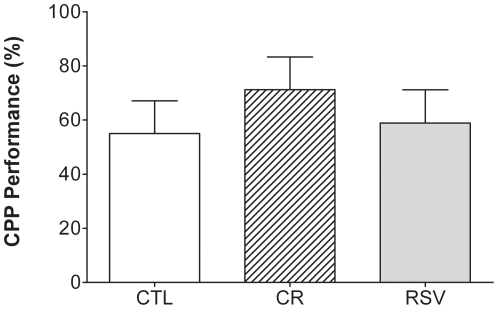
Effects of CR and RSV treatments on percentage of reversal in a conditioned placed preference (CPP) task. Data of control (CTL, n = 8), caloric restricted (CR, n = 8) or resveratrol supplemented (RSV, n = 9) mouse lemurs after 18 months of dietary interventions are reported. Values are mean ± SEM.

CPP performance expressed as the percentage of reversal (Fig. 3) was 55±11% in CTL animals and was not significantly different in CR (74±13%, U = 42, p = 0.249) and RSV supplemented animals (55±12%, U = 39.5, p = 0.929).

### 4 OF task

**Figure 4:**
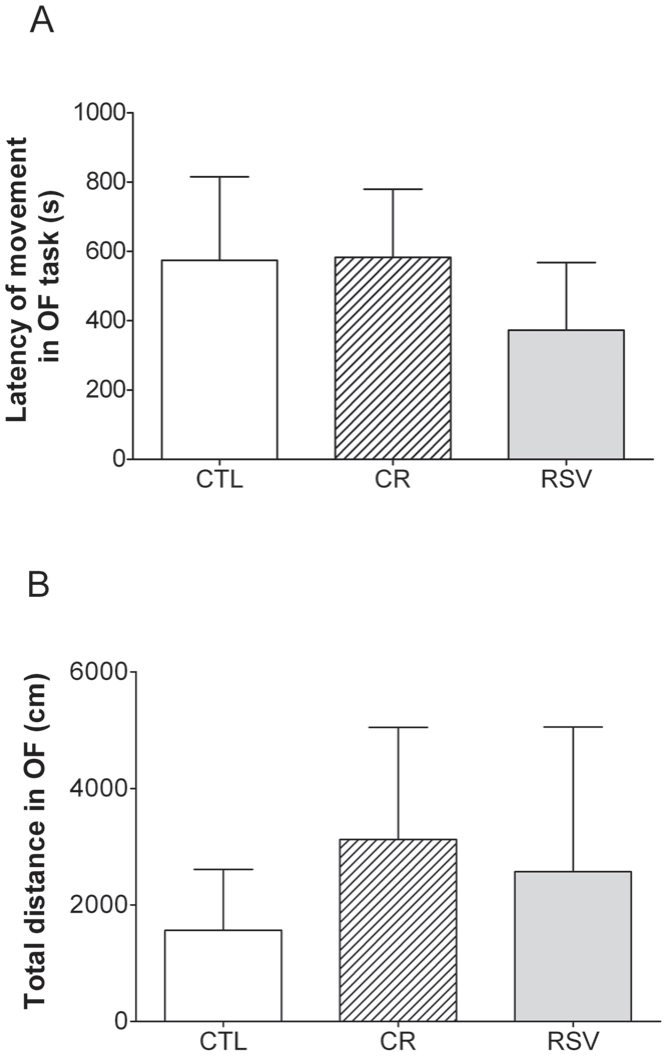
Effects of CR and RSV treatments on cognitive performances in an open field (OF) task. Figure 4A and 4B respectively represent the latency (expressed in sec) before the first movement in the open field and the total distance traveled (expressed in cm) in the open field task of control (CTL, n = 9), caloric restricted (CR, n = 9) or resveratrol supplemented (RSV, n = 10) mouse lemurs after 18 months of dietary interventions. Values are mean ± SEM.

No significant difference was found between CR *vs* CTL (448±159 s and 302±193 s, U = 54, p = 0.943) and RSV *vs* CTL (87±41 s and 302±193 s, U = 64, p = 0.901) for the latency of first movement which averaged 272±84 s (Fig. 4A). Similar results were obtained for the total distance travelled by the animals in OF task with no significant difference between CR *vs* CTL (3469±2117 cm and 1911±1261 cm, U = 68, p = 0.359) and RSV *vs* CTL (3084±2974 cm and 1911±1261 cm, U = 75, p = 0.579) (Fig. 4B, mean  = 2831±1238 cm).

### 5 Rotarod task, jump task and spontaneous night locomotor activity

**Figure 5:**
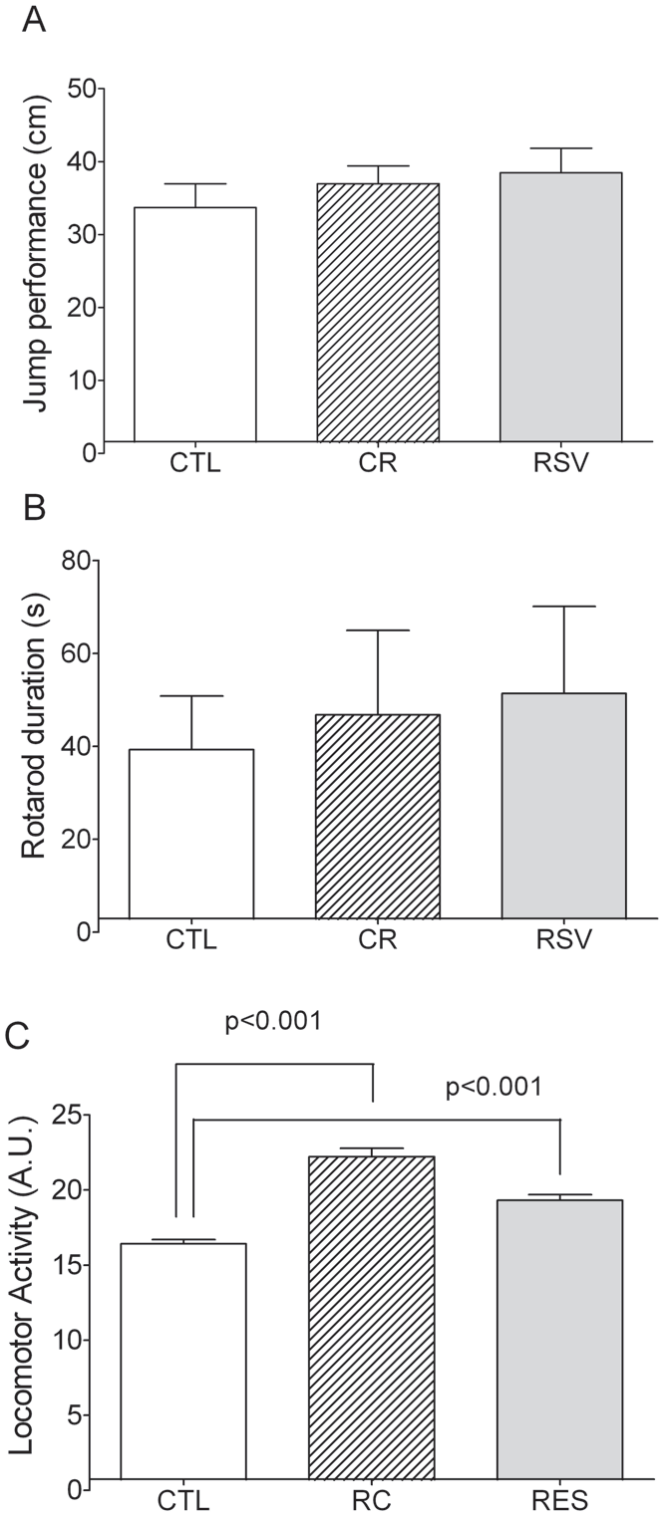
Effects of CR and RSV treatments on physical performances in motor tasks and spontaneous night locomotor activity. Data of control (CTL), caloric restricted (CR) or resveratrol supplemented (RSV) mouse lemurs after 18 months of dietary interventions are reported. Figure 5A represents physical performances of the animals in a jump task (expressed in cm, with n = 9 CTL, n = 11 CR and n = 10 RSV). Figure 5B represents physical performances of the animals in rotarod task (expressed in sec, with n = 7 CTL, n = 9 CR and n = 8 RSV). Figure 5C represents spontaneous night locomotor activity (expressed in arbitrary units of locomotor activity, with n = 7 CTL, n = 6 CR and n = 7 RSV). Values are mean ± SEM.

No significant difference was found between CR *vs* CTL (U = 30.5, p = 0.875) and RSV *vs* CTL (U = 30.5, p = 0.875) for the physical performances in the rotarod task (Fig. 5A, mean  = 43.4±9.5 s) In the same way, no significant difference was observed between CR *vs* CTL (U = 56.5, p = 0.603) and RSV *vs* CTL (U = 39.5, p = 0.401) for the physical performances in the jump task (Fig. 5B, mean  = 35.0±1.7 cm). Moreover, no correlation appeared between Rotarod or jump performances and body mass of mouse lemurs (r = −0.475, *n* = 24, NS, and r = −0.132, *n* = 30, NS, respectively). CR animals exhibited a significantly higher night locomotor activity (21.5±0.6 a.u.) compared to CTL (15.7±0.3 a.u.) (U = 34, p = 0.043). RSV supplemented animals also had a significantly higher night locomotor activity (18.7±0.4 a.u., U = 13, p = 0.042) compared to CTL animals.

### 6 Urinary cortisol level

**Figure 6:**
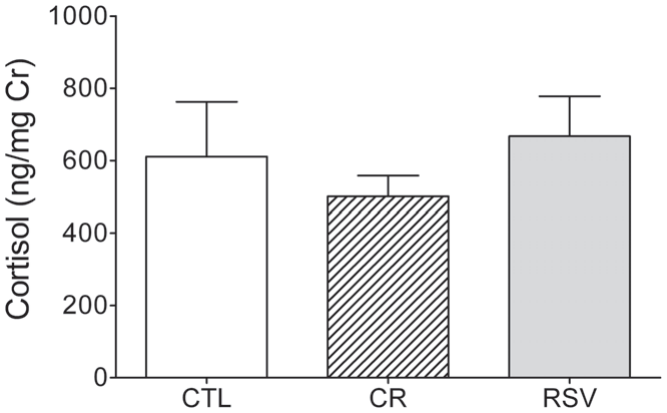
Effects of CR and RSV treatments on cortisol level (expressed in ng/mg of creatinine). Data of control (CTL, n = 9), caloric restricted (CR, n = 10) or resveratrol supplemented (RSV, n = 11) mouse lemurs after 18 months of dietary interventions are reported. Values are mean ± SEM.

The assessment of urinary cortisol level revealed no significant difference between CR *vs* CTL (U = 44, p = 0.935) and RSV *vs* CTL (U = 40, p = 0.470) (Fig. 6). Urinary cortisol level was 611±143 ng/mg of creatinine in CTL animals and 507±63 and 667±111 ng/mg of creatinine in the CR and RSV supplemented animals, respectively.

## Discussion

A cohort of adult grey mouse lemurs was followed in order to evaluate their cognitive and motor performances as well as their emotionality after 18 months of chronic moderate CR or RSV supplementation. Regarding the impact of CR on behaviour, many of the previous studies from rodents and humans showed controversial effects. In rodents, CR has been described as beneficial for cognitive performances in mice [12], [39] and mainly deleterious in rats [13], [14]. In humans a 3-month 30% caloric restriction led to a significant increase in verbal memory scores and was correlated with decreased fasting plasma levels of insulin and high sensitive C-reactive protein [2]. Accordingly to what was observed in humans, we expected positive effects of mid-life onset CR on cognitive functions in this primate model. Very few studies reported information about RSV effect on cognitive function. For example, Kumar et al. showed that administration of resveratrol in rats that received an intracerebroventricular colchicine injection, known to cause loss of cholinergic neurons and cognitive dysfunction that is associated with excessive free radical generation, had a neuroprotective role against colchicine-induced disturbances [40]. Moreover, Joseph et al. demonstrated that resveratrol and more particularly, one of its most efficacious analogue (pterostilbene) were effective in reversing cognitive behavioral deficits, as well as dopamine release, in aged rats and their working memory was correlated with pterostilbene levels in the hippocampus. These previous results allowed us to expect beneficial effects of this polyphenol on memory performances of mouse lemur. In the present report we show that both CR and RSV supplementation were accompanied by maintained or better cognitive and motor performances after 18 months of chronic treatment. Main effects of treatments were observed in the continuous spontaneous alternation task, in which both CR and RSV supplemented animals presented better performances of total alternation compared to CTL animals. RSV supplementation also significantly lowered the number of errors in the circular platform task compared to CTL whereas CR did not significantly change the performances to this test.

Animals of the three groups have comparable motor performances (jump and rotarod tasks) suggesting that both treatments and more particularly CR, did not impair the motor functions of the animals. These two motor tasks require strength, spring and suppleness and mobilize a high amount of energy and all animals were able to achieve them, showing their good general health condition. Since motor performances are not changed with treatments, we can exclude that changes observed in cognitive tasks could be due to motor modifications. On the other side, animals under CR exhibited a significantly increased spontaneous night locomotor activity (LA) with a noteworthy mimetic effect of RSV. Since this increased activity occurs during night, it can not be attributed to a food anticipatory activity that has been described to occur during the day in other food deprivation protocols [41]. A previous study already showed that old rhesus monkeys (*Macaca mulatta*) under a 30% CR presented an increase in spontaneous activity levels [42], what is also consistent with previous observations made in rodents under CR [43]. Since changes in night LA can not be linked to modified motor functions and, since the amplitude of LA rhythms between night and day increased, we can hypothesise that both CR and RSV exert stimulatory effects on circadian clock. Such a property deserves to be further investigated.

We found no evidence of any effect of CR treatment after 18 months of treatment on behavioural performances in the open field task and cortisol levels. Thus, CR treatment, conversely to what observed in mice [12], did not induce more anxiety in CR animals compared to CTL. Comparable findings where also observed under RSV supplementation.

As a spatial reference memory task requiring animals to remember a specific location, the circular platform test is sensitive to hippocampus integrity. Thus, our results showed no deleterious impact of CR on the spatial memory of the mouse lemurs after 18 months of treatment. Indeed, CR animals did not make more errors to find the exit compared to CTL animals and both groups performed the same time to reach the exit in the circular platform task. These results suggest that the CR animals acclimated physiologically to their imposed diet. However, a negative effect of CR on spatial cognition was reported in rats by Yanai et al. using the Morris water maze [14]. Given that injection of glucose improved performance of the CR group to the level of the *ad libitum* group, this negative impact of CR was interpreted by the authors as a reduced glucose availability in the hippocampus for the CR animals. This observation allows us to hypothesize that in CR mouse lemurs a re-allocation of energy distribution to the brain could occur, thereby suppressing a potential deleterious effect of CR on hippocampus-dependent spatial memory task. This hypothesis should be confirmed in further studies in which cognitive tests will be carry out after only a short period of treatment. On the other side, the significantly lower number of errors of RSV supplemented animals in this spatial reference memory task suggested a positive effect of RSV on hippocampus activity or functions. If the mechanism of RSV impacts on brain functions remain unexplored in the mouse lemur, it is widely described that RSV increases the activation of sirtuin 1 (SIRT1), a NAD(+)-dependent deacetylase [44] implied in energy metabolism regulation. Improvement of energy utilisation under RSV supplementation could lead to better specific brain functions.

In the conditioned preference place task, measuring the emotional memory mediated by the amygdala, animals under both treatments exhibit comparable performances compared to CTL. This task was designed to assess learning of associations between a distinctive environment or “place” and appetitive or aversive stimuli. Such learned associations result in approach or avoidance behaviors with respect to these places. There is considerable evidence that the amygdala is fundamentally involved in such reward-stimulus or punishment-stimulus associations [36], [45]. However, amygdala-dependent emotional memory tasks are known to be well preserved during normal aging in human [46], [47] and the grey mouse lemurs followed here were adults. Ours results corroborate well with the previous observations. Potential effects of both treatments on this brain structure would be probably observed in older mouse lemur but further investigations will be necessary to confirm this hypothesis.

Both dietary treatments led to significantly better performances of total alternation in the continuous spontaneous alternation task than that displayed by the CTL group without changes in partial alternation. This difference between partial and total alternation performances could be explained by the fact that partial alternation is easier to perform for the animals than total alternation, hence the lack of effect in the first task. For total alternation, it is reasonable to hypothesize that similar to CR treatment, RSV supplementation stimulates cognitive functions involved in this continuous spontaneous alternation task. Besides requiring motivation to explore, spontaneous alternation performance critically depends on working memory and strategy efficiency. Such strategy requires precise memory of the temporal order of visited arms. Working memory and flexible strategy are parts of executive functions fundamentally subserved by striato-prefrontal circuits [48], [49], [50]. Positive effects of RSV observed in this study could be explain by its antioxidant properties that provides neuroprotective effects [19], [22] and by its capacities to stimulate cholinergic transmission and consequently improve cognition [20]. Given the central role of cholinergic neurotransmission in attentional processing [51], [52], RSV treatment could therefore have a positive impact on task involving executive and memory functions that underlie controlled behaviours requiring a high level of attention.

In conclusion, cognitive performances of adult mouse lemurs under chronic CR are maintained or improved in the case of executive functions. Moreover, the present results show, for the first time in an adult primate, a positive effect of RSV on cognitive function (in both executive function and spatial memory). CR and RSV treatments seemed to induce similar benefits on cognitive functions by probably activating similar brain structures (striato-prefrontal circuits and hippocampus) and have similar effects on locomotor activity. These observations allow us to suggest that RSV could be a good candidate to mimic long-term CR effects. The present results obtained in adult animals are promising with regard to possible positive effects during aging and also support the growing evidences that nutritional interventions can have beneficial effects on brain functions even in adults.
